# Clinical effect of nighttime snacking on patients with hepatitis B cirrhosis

**DOI:** 10.3389/fnut.2022.999462

**Published:** 2023-01-10

**Authors:** Zuoqing Han, Rongkuan Li, Zhiwei Zhong, Yuetong Piao, Rong Guo

**Affiliations:** ^1^Graduate School, Dalian Medical University, Dalian, China; ^2^Department of Infection, The Second Hospital of Dalian Medical University, Dalian, China

**Keywords:** liver cirrhosis, improving quality of life, hepatitis B cirrhosis, clinical effect, night snacking

## Abstract

**Objective:**

Nighttime snacking is an effective intervention to avoid abnormal protein consumption caused by prolonged fasting. This article aims to evaluate the clinical efficacy of nighttime snacking on patients with hepatitis B cirrhosis and to provide new ideas for clinical nutritional intervention.

**Methods:**

The study participants were randomly assigned to the control group (*n* = 30) and the observation group (*n* = 30); the former was administered medical system treatment and routine dietary intervention, and the latter was administered the same treatment with the addition of nighttime snacking. After 3 months of intervention with different dietary guidance, the dry body weight body mass index (BMI), upper arm muscle circumference (AMC), grip strength, triceps skinfold thickness (TSF), third lumbar skeletal muscle index (L3-SMI), albumin (ALB), total bilirubin (T-BIL), cholinesterase (CHE), Fried's frailty phenotype score, Child–Pugh score and various cirrhosis complication rates were compared between the two groups.

**Results:**

There was no significant difference in the baseline data between the two groups before the dietary intervention. After 3 months of regular dietary guidance in the control group, the grip strength increased compared with the baseline data (*p* < 0.05), while the dry body weight BMI, AMC, TSF, L3-SMI, ALB, T-BIL, CHE, prothrombin time, international normalized ratio, prothrombin activity, and Child–Pugh scores were not significantly different (*p* > 0.05). After 3 months of dietary guidance with nighttime snacking in the observation group, the dry body weight BMI, grip strength, TSF, L3-SMI, and CHE scores all increased, compared with the baseline data, while the Child–Pugh score decreased compared with the baseline level (all *p* < 0.05). After 3 months of intervention, the Child–Pugh score of the observation group showed a more significant decrease than the control group, while the dry body weight BMI, grip strength, ALB and CHE scores were all significantly higher than those in the control group (all *p* < 0.05). Overall, the improvement rate was significantly higher in the observation group than in the control group (*p* < 0.05).

**Conclusion:**

Nighttime snacking for hepatitis B cirrhosis patients with nutritional risk is beneficial in terms of the recovery of liver synthesis functions, improvements in clinical indicators, sarcopenia corrections and improvements in malnutrition-related complications.

## 1. Introduction

It is estimated that there are currently 7 million patients with liver cirrhosis in China (1). Among them, the annual mortality rate of patients with decompensated liver cirrhosis can reach 20–55% (1). Patients with liver cirrhosis have poor glycogen reserves and abnormal metabolism of energy substrates, meaning their states of starvation after 10 h are comparable to starvations lasting 2–3 days for healthy individuals (2). Nighttime snacking is an effective intervention to avoid abnormal protein consumption caused by prolonged fasting (3). At present, domestic and foreign guidelines recommend that patients with liver cirrhosis should not undergo more than 12 h of fasting, and that their daily calorie intake should be divided into 4–6 meals, including nighttime snacks (1, 2). Nighttime snacking has been proven to improve the energy metabolism, hepatic functional reserve and sarcopenia in patients with cirrhosis, all of which increase the patients' chances of survival (3, 4).

Relevant nutrition guidelines in China and the United States have recommended nighttime snacking as a reasonable and effective dietary pattern for patients with liver cirrhosis. Conversely, there are currently few relevant studies in China, and clinicians are uncertain about whether to recommend the consumption of midnight snacks to their patients. Therefore, this study assesses the nutritional status of a group of patients with hepatitis B cirrhosis and analyses the issues pertaining to providing guidance for the patients to implement late-night snacking in clinical applications. Overall, this study provides a data-driven basis for clinicians seeking to guide their patients regarding the consumption of nighttime snacks.

## 2. Materials and methods

### 2.1. Research participants

This study included a total of 60 patients with hepatitis B cirrhosis who were diagnosed in the Second Affiliated Hospital of Dalian Medical University from January 2020 to December 2021. The inclusion criteria were as follows: (1) patients who met the diagnostic criteria for hepatitis B cirrhosis recorded in the *Guidelines for the Prevention and Treatment of Chronic Hepatitis B* (2019 Edition) (5); (2) patients who met the nutritional risk screening 2002 score (NRS-2002, score ≥ 3); and (3) patients who agreed to participate in this study and who signed an informed consent form. The exclusion criteria were as follows: (1) patients with a liver cirrhosis etiology that differed from hepatitis; (2) patients with hyperthyroidism, a severe infection, renal insufficiency, malignant tumors other than liver tumors or drugs that clearly affect metabolism (e.g., glucocorticoids); (3) patients with advanced liver cancer and patients with metastatic recurrence; (4) patients with mental disorders who could not cooperate; and (5) patients who were unwilling to participate in the study. The elimination criteria during the research process included the following: (1) voluntary requests to withdraw from the research; (2) patients in the control group who changed their eating habits and consumed nighttime snacks without authorization; (3) patients in the observation group who did not consistently consume nighttime snacks; and (4) patients who did not adhere to the follow-up for various reasons.

This study was approved by the Ethics Committee of the Second Affiliated Hospital of Dalian Medical University, and all participants signed an informed consent form. Based on the random number table method, the patients who met the inclusion and exclusion criteria were randomly divided between the control and observation groups. Excel software was used to generate 60 random numbers, which were then sorted to give numbers 1–30 for the intervention group and numbers 31–60 for the control group.

### 2.2. Research methods

Nutrition education was provided to the enrolled patients and their families; the patients were instructed on the dietary principles of liver cirrhosis using 1-day recipes as examples. The patients were also taught how to calculate the energy and protein contents of the foods using the “Chinese Food Composition Table,” which can provide lists of the nutrients in various foods based on weight. The two groups of patients were instructed to achieve a recommended daily intake of total energy (30–35 kcal/kg^−1^·day^−1^) and protein (1.2–1.5 g/kg^−1^·day^−1^). The patients in the observation group were asked to reduce their intake of carbohydrate-based foods by 100 kcal at both lunch and dinner and to consume a snack consisting of a total of 200 kcal (100 kcal × 2) in carbohydrate-based foods (such as lotus root starch, bread and noodles) 30 min^−1^ h before going to bed. All patients were instructed to eat and drink according to the nutrition lectures and to record all food and drink consumed each day within 24 h of consumption (from midnight to midnight). The researchers conducted follow-ups *via* WeChat or telephone every 10 days, and the patients were asked to provide dietary records at those times. The total intervention period was 3 months. Both groups of patients were advised to maintain their usual activity levels during the intervention period.

### 2.3. Data collection

All of the patients were fully evaluated before and after the intervention with detailed medical history records, abdominal computed tomography (CT), magnetic resonance imaging (MRI), venous blood sample collections and blood biochemical markers.

#### 2.3.1. Anthropometric indicators

The body mass index (BMI) (weight/height squared [kg/m^2^]), and dry body weight BMI (dry weight/height squared [kg/m^2^]) was calculated for each patient experiencing fluid retention. The upper arm circumference (AC) was measured using a soft ruler, with the reading accurate to within 0.1 cm, while the triceps skinfold thickness (TSF) was measured using a sebum thickness meter, with the reading accurate to within 0.1 mm. The arm muscle circumference (AMC) was measured in terms of AC (cm) – 3.14 × TSF thickness (cm), and the grip strength was measured using an electronic grip dynamometer. The measurement of the third lumbar vertebra skeletal muscle index (L3-SMI) involved selecting the middle layer of the L3 from the abdominal CT and MRI images (the horizontal transverse process of this layer is symmetrical and clearly visible) using sliceOmatic 5.0 image analysis software. All muscles of this level image were identified and calibrated using the Hounsfield unit threshold value of −29 to +150, and all skeletal muscles in this plane (psoas major, erector spinae, quadratus lumborum, transverse abdominis, external oblique muscle, and abdominal sum of the cross-sectional areas of the internal oblique and rectus abdominis) were divided by the square of the height to obtain the L3-SMI (cm^2^/m^2^), with the measurements accurate to within two decimal places.

#### 2.3.2. Detection of blood biochemical indicators

The blood biochemical indexes of each group of patients were evaluated by the laboratory department of the Second Affiliated Hospital of Dalian Medical University before and after the intervention. The evaluation indicators included the following liver biochemistry factors: prothrombin time (pT, s), international normalized ratio (INR), prothrombin activity (pTA, %), and the serum cholinesterase (CHE), albumin (ALB) and total bilirubin (T-BIL) levels.

#### 2.3.3. Clinically relevant indicators

The evaluation criteria of clinical indicators were the Child–Pugh scores before and after intervention, the incidence of new complications (Fried's debilitating phenotype) related to malnutrition and the improvement rate of any existing complications. The complications associated with cirrhosis include spontaneous peritonitis, hepatic encephalopathy, upper gastrointestinal bleeding, ascites, electrolyte disturbances and hepatorenal syndrome. The diagnosis and severity assessments of these complications were performed in accordance with the relevant guidelines (6, 7). The other evaluation criteria included any new complications associated with malnutrition (Fried's debilitating phenotype) (8, 9).

### 2.4. Follow-up

During the intervention period, one-to-one health education and dietary counseling were given to 69 subjects through WeChat, and their dietary intakes were assessed weekly using a 24-h dietary review method. The researchers also conducted dietary follow-ups every 10 days to analyse the effects of the diet on the patients, to obtain their experiences and to provide encouragement and psychological guidance. If the subjects experienced any discomfort or doubts during the dietary guidance process, they could communicate directly with the researchers through WeChat. After 3 months, the relevant indicators of the outpatient or in-hospital re-examinations were assessed to evaluate individual patient conditions, and the follow-ups were completed.

### 2.5. Statistical methods

The data were analyzed using SPSS 23.0 statistical software. The measurement data that satisfied the normal distribution were expressed as means ± standard deviations, with the independent samples *t-*test used for the inter-group comparisons, and the paired samples *t-*test used to assess the changes within each group before and after the intervention, if the normal distribution was satisfied.

The measurement data and grade data that did not meet the normal distribution and/or did not have homogeneity of variance were expressed in terms of median (interquartile) range, with an independent sample non-parametric test used for the inter-group comparisons. If the normal distribution was not satisfied, a paired sample non-parametric test was used. The enumeration data were expressed as rate (%) and percentage using a chi-square/Fisher's test. The repeated measures data were analyzed using the repeated measures analysis of variance method. All of the results of the above statistical analyses were statistically different at *p* < 0.05.

The statistical power was examined using PASS 15 statistical software (NCSS, LLC, Kaysville, Utah, USA). The power for the primary endpoint (improvement rate after 3 months) was calculated based on a two-sided chi-square test with a significance level of 5%. With a final sample size of 15 patients in the observation group and 19 patients in the control group, the trial entailed an 84.8% statistical power to detect the difference between the two groups, given that the improvement rate of cirrhosis following the 3-month intervention was 46.7% for the observational group and 10.5% for the control group. Specifically, the 84.783% power allowed for detecting a difference between the groups of 0.3620. The proportion in the treatment group was assumed to be 0.1050 under the null hypothesis and 0.4670 under the alternative hypothesis, while the proportion in the control group was 0.1050. The test statistic used was the two-sided *Z*-test with un-pooled variance. The significance level of the test was 0.1500.

## 3. Results

In this study, a total of 73 patients with hepatitis B cirrhosis met the inclusion criteria, and 69 patients agreed to participate in this study and completed the randomization process and the baseline data collection. A final total of 60 patients completed the entire study observation. There were 30 cases in the observation group and 30 cases in the control group. During the 3-month intervention, 5 subjects in the observation group withdrew from the study; 4 withdrew because they could not adhere to the nighttime snacking, and 1 withdrew during the follow-up. Additionally, 4 subjects in the control group withdrew from the study; 2 failed to comply with the study protocol and 2 withdrew during the follow-up. There was no significant difference in the drop-off rate between the two groups (*p* > 0.05), and the withdrawals did not have a significant impact on the study results.

### 3.1. Comparison of the demographics and baseline parameters of the two groups

The mean age of the patients in the observation group was 57.07 ± 9.44 years, with 21 male patients (70.0%). The mean age of the patients in the control group was 54.83 ± 12.57 years, with 22 male patients (73.7%). The scores for age, gender, dry weight BMI, ALB, T-BIL, CHE, grip strength, AMC, TSF, L3-SMI, Child–Pugh score, incidence of complications of liver cirrhosis, proportion of interventional treatment, incidence of ascites in the two groups, ascites severity and Fried's frailty phenotype were not significantly different (*p* > 0.05), and the indicators were consistent between the two groups (Table 1).

**Table 1:** Comparison of the general conditions of the observation group and the control group before the intervention.

**Title**	**Control group (*n* = 30)**	**Observation group (*n* = 30)**	**Statistics**	***p-*value**
Age (years)	54.83 ± 12.57	57.07 ± 9.44	*t* = 0.778	0.439
Male (case, %)	22 (73.7)	21 (70.0)	χ^2^ = 0.082	0.774
Dry weight BMI (kg/m^2^)	24.09 (21.60, 26.33)	21.91 (20.58, 25.46)	*Z* = −1.175	0.240
Albumin (g/L)	37.99 ± 6.42	38.42 ± 5.99	*t* = 0.27	0.788
Total bilirubin (μmol/L)	22.19 (17.19, 36.14)	17.84 (14.75, 28.47)	*Z* = −1.508	0.132
Cholinesterase (U/L)	5,349.9 (2,996.5, 6,540.3)	5,843 (3,335, 6,718.8)	*Z* = −0.458	0.647
L3-SMI (cm^2^/m^2^)	45.73 (40.33, 51.02)	43.55 (38.32, 49.21)	*Z* = −1.028	0.304
Grip (kg)	33.46 ± 6.70	32.88 ± 4.96	*t* = −0.381	0.704
AMC (mm)	23.35 (21.68, 25.12)	22.40 (20.99, 24.41)	*Z* = −1.368	0.171
TSF (mm)	13.86 ± 2.26	13.42 ± 2.23	*t* = −0.747	0.458
PCI (case, %)	17 (56.7)	14 (46.7)	χ^2^ = 0.601	0.438
Ascites (case, %)	12 (40.0)	16 (53.3)	χ^2^ = 1.071	0.301
Child–Pugh score (point)	6 (5, 9)	7 (6, 8.25)	*Z* = −0.972	0.331
Liver cirrhosis complications (case, %)	19 (63.3)	15 (50.0)	χ^2^ = 1.086	0.297
Severity of ascites (case, %)			χ^2^ = 1.8	0.615
NO	18 (60.0)	14 (46.7)		
Mild	6 (20.0)	9 (30.0)		
Medium	3 (10.0)	5 (16.7)		
Severe	3 (10.0)	2 (7.7)		
Fried frail phenotype (cases, %)			χ^2^ = 1.227	0.54
No Frailty	11 (36.7)	14 (46.7)		
Pre-frailty	12 (40.0)	8 (26.7)		
Frailty	7 (23.3)	8 (26.7)		

BMI, body mass index; L3-SMI, third lumbar skeletal muscle index; AMC, upper arm muscle circumference; TSF, triceps skinfold thickness; PCI, percutaneous coronary intervention.

### 3.2. Comparison of the clinical indicators in the observation group after 3 months of nighttime snacking dietary guidance

Compared with the baseline data, the dry body weight BMI, TSF, grip strength, L3-SMI, and CHE of the observation group all increased, while the Child–Pugh score decreased compared with the baseline level, with significant differences (*p* < 0.05); there were no significant differences in terms of AMC, ALB, and T-BIL (*p* > 0.05) (Table 2).

**Table 2:** Comparison of clinical indicators in observation group after 3 months of nighttime snack dietary guidance.

**Title**	**Before dietary guidance**	**After dietary guidance**	**Statistics**	***p*-value**
Dry weight BMI (kg/m^2^)	21.91 (20.58, 25.46)	23.28 (21.40, 25.83)	*Z* = −2.56	0.011
TSF (mm)	13.42 ± 2.23	14.24 ± 2.34	*t* = −3.390	0.002
AMC (mm)	22.4 (20.99, 24.41)	23 (21.45, 25.05)	*Z* = −1.449	0.147
Grip (kg)	31.33 (28.94, 36.89)	37.05 (32.95, 41.2)	*Z* = −4.054	<0.001
L3-SMI (cm^2^ /m^2^)	43.55 (38.32, 49.21)	48.21 (38.63, 55.55)	*Z* = −2.149	0.032
Albumin (g/L)	38.42 ± 5.99	39.45 ± 6.49	*t* = −1.645	0.111
Total bilirubin (μmol/L)	17.84 (14.75, 28.47)	18.27 (14.63, 26.23)	*Z* = −0.463	0.644
Cholinesterase (U/L)	5,843 (3,335, 6,718.75)	6,141.5 (3,815.5, 7,585.5)	*Z* = −2.733	0.006
Child–Pugh score (Point)	7 (6, 8.25)	6 (5, 7)	*Z* = −3.469	0.001

BMI, body mass index; TSF, triceps skinfold thickness; AMC, upper armmuscle circumference; L3-SMI, third lumbar skeletal muscle index.

### 3.3. Comparison of the clinical indicators in the control group after 3 months of regular dietary guidance

After 3 months of regular dietary guidance in the control group, the grip strength increased compared with the baseline data with a significant difference (*p* < 0.05), while there was no significant difference in terms of pT, INR, pTA or Child–Pugh score (*p* > 0.05) for the dry body weight BMI, TSF, AMC, L3-SMI, ALB, T-BIL, and CHE (Table 3).

**Table 3:** Comparison of clinical indicators in the control group after 3 months of regular dietary guidance.

**Title**	**Before dietary guidance**	**After dietary guidance**	**Statistics**	***p*-value**
Dry weight BMI (kg/m^2^)	24.09 (21.60, 26.33)	24.10 (20.94, 26.17)	*Z* = −0.071	0.943
TSF (mm)	13.86 ± 2.26	14.17 ± 2.38	*t* = −1.123	0.271
AMC (mm)	23.35 (21.68, 25.12)	24.5 (21.9, 25.5)	*Z* = −0.261	0.794
Grip (kg)	34.90 (27.69, 39.33)	35 (28.55, 42.13)	*Z* =-2.266	0.023
L3-SMI (cm^2^ /m^2^)	45.73 (40.33, 51.02)	46.70 (38.68, 51.54)	*Z* = −0.422	0.673
Albumin (g/L)	37.99 ± 6.42	37.19 ± 6.21	*t* = 1.315	0.199
Total bilirubin (μmol/L)	22.19 (17.19, 36.14)	24.89 (15.63, 36.67)	*Z* = −0.697	0.486
Cholinesterase (U/L)	5,349.9 (2,996.5, 6,540.25)	5,214.9 (3,058.75, 6,723)	*Z* = −1.063	0.288
Prothrombin time (s)	14.75 (13.58, 15.7)	14.35 (13.8, 15.43)	*Z* = −0.747	0.455
INR	1.16 (1.06, 1.26)	1.15 (1.06, 1.22)	*Z* = −0.107	0.915
PTA (%)	78.5 (72.25, 90.75)	80.5 (72.75, 90.5)	*Z* = −0.115	0.909
Child–Pugh score (point)	6 (5, 9)	6 (5.75, 8)	*Z* = −0.288	0.773

BMI, body mass index; TSF, triceps skinfold thickness; AMC, upper arm muscle circumference; L3-SMI, third lumbar skeletal muscle index; INR, international normalized ratio; PTA, prothrombin activity.

### 3.4. Comparison of the clinical indicators between the two groups after 3 months of dietary guidance

The differences between the indicators before and after the intervention were compared in terms of the two groups. The results indicated that the Child–Pugh score of the observation group decreased more than that of the control group, while the enhancement in ALB, the upsurge in dry body weight BMI, the increase in grip strength and the increase in CHE were higher than those of the control group, with the differences statistically significant (*p* < 0.05). However, there were no significant differences in terms of AMC, L3-SMI, T-BIL, and TSF (*p* > 0.05) (Table 4).

**Table 4:** Comparison of differences in clinical indicators between the two groups of subjects after 3 months of dietary guidance.

**Title**	**Observation group (*n* = 30)**	**Control group (*n* = 30)**	**Statistics**	***p*-value**
Difference in dry weight BMI (kg/m^2^)	0.67 (0, 1.24)	0 (−0.71, 0.06)	*Z* = −2.29	0.022
Total bilirubin difference (μmol/L)	−0.07 (−6.99, 4.41)	0.68 (−3.53, 5.28)	*Z* = −0.732	0.464
AMC difference (mm)	0.25 (−0.62, 1.33)	0 (−0.6, 0.6)	*Z* = −0.704	0.481
L3-SMI difference (cm^2^/m^2^)	0.81 (−0.57, 15.38)	0.44 (−3.19, 2.72)	*Z* = −1.412	0.158
Cholinesterase difference (U/L)	379.5 (0, 1,017)	−4.95 (−372, 224.25)	*Z* = −3.181	0.001
Grip strength difference (kg)	3.53 (0.52, 6.55)	0.35 (−0.35, 4.08)	*Z* = −2.432	0.015
TSF difference (mm)	0.83 ± 1.33	0.31 ± 1.51	*t* = −1.399	0.167
Albumin difference (g/L)	1.03 ± 3.43	−0.79 ± 3.33	*t* = −2.096	0.04
Child–Pugh score difference (points)	−1 (−2, 0)	0 (0, 0.25)	*Z* = −3.145	0.002

BMI, body mass index; AMC, upper arm muscle circumference; L3-SMI, third lumbar skeletal muscle index; TSF, triceps skinfold thickness.

### 3.5. The improvement in liver cirrhosis complications after 3 months of dietary guidance in the two groups

Before the nutrition intervention, 15 patients in the observation group and 19 patients in the control group had complications. After 3 months of dietary guidance, 7 patients (46.7%) in the observation group had improved complications. Specifically, 2 patients had spontaneous peritonitis, 3 had electrolyte imbalances, 1 had hepatic encephalopathy and 1 had upper gastrointestinal bleeding. In the control group, 2 patients (10.5%) had improved complications, 1 with spontaneous peritonitis and 1 with hepatic encephalopathy. There was a statistically significant difference in the improvement in complications between the two groups (*p* < 0.05) (Table 5). Details of the remission of the complications in the two groups after 3 months of dietary guidance are presented in Supplementary Tables 1, 2.

**Table 5:** Improvement of liver cirrhosis complications after 3 months of dietary guidance in the two groups.

**Group**	**Total (case)**	**Better [case (%)]**	**No change or otherwise [case (%)]**	***p*-value**
Observation group	15	7 (46.7)	8 (53.3)	0.025
Control group	19	2 (10.5)	17 (89.5)	

One or more complications can exist in the same patient at the same time and improvement of one of them is regarded as improvement.

### 3.6. Nutritional screening and assessment

We used the NRS-2002 score, Global Leadership Initiative on Malnutrition (GLIM) criteria and the Royal Free Hospital–Nutritional Prioritizing Tool (RFH-NPT) to assess the nutritional status of the two groups of patients. According to the GLIM criteria, the prevalence of malnutrition was 70.00% (21/30) in the observation group and 70.00% (21/30) in the control group. Specific data are listed in Supplementary Table 3. According to the RFH-NPT, the prevalence of malnutrition was 80.00% (24/30) in the observation group and 70.00% (21/30) in the control group. Specific data are listed in Supplementary Table 4. There was no significant difference between the two groups.

## 4. Discussion

Chronic hepatitis B virus infections in patients with hepatitis B cirrhosis lead to impaired liver function, which, in turn, affects the synthesis of three major nutrients. At the same time, energy intake and metabolic disorders, as well as low body resistance caused by disturbances in the body's internal environment, will further reduce protein synthesis and ultimately lead to malnutrition. When patients suffer from malnutrition, liver damage is further aggravated, the risk of complications is increased and the malnutrition is progressively exacerbated, resulting in a vicious circle (10, 11).

The current study results indicated that after 3 months of nutritional guidance, the Child–Pugh scores and the improvement rates, in terms of complications, were higher in the observation group than in the control group. There was a significant incidence of frailty and pre-frailty in the hepatitis B cirrhosis patients included in the study, with 53.4% in the observation group and 63.3% in the control group. After 3 months of dietary intervention, the Fried's frailty phenotype scores decreased in both groups; however, the changes were more significant in the observation group, which may have been related to the improvement in the anthropometric indicators reflecting skeletal muscle strength and muscle mass.

Professor Rosenberg first proposed the definition of sarcopenia in the 1980s. In brief, sarcopenia is a syndrome caused by continuous loss of skeletal muscle mass, strength and function. The skeletal muscle is the driving force of the human motor system; the atrophy of this muscle is a major sign of human aging and can easily result in fractures and joint injuries. Older people with sarcopenia have difficulty standing and walking and are prone to falls that lead to fractures. The condition can also affect organ functioning, potentially leading to heart and lung failure, and even death. Sarcopenia includes two aspects: skeletal sarcopenia and muscle dysfunction (12), with the former a recognized complication of cirrhosis that is associated with an increased risk of complications, poor quality of life and a higher risk of mortality while waiting for transplantation or following transplantation (13, 14). Meanwhile, muscle dysfunction results in a significant decrease in muscle strength. The attendant treatment options are limited and are focused primarily on the lack of replacement rather than on targeting the underlying cause; options for treatment include nutritional supplementation, exercise, combined lifestyle interventions, testosterone replacement and transjugular intrahepatic portosystemic shunts to improve the muscle mass in cirrhosis patients (15).

The pathogenesis of sarcopenia in cirrhosis is multifactorial. For patients with liver cirrhosis, malnutrition is also one of the important causes of the condition, largely due to systemic inflammation, physical inactivity and specific environmental factors (16). A low-sodium diet leads to malnutrition secondary to ascites, delayed gastric emptying, and decreased appetite and palatability, resulting in reduced food intake and imbalanced muscle synthesis and degradation (17).

The L3-SMI can directly reflect the state of human muscle mass without the influence of body fluid retention, and its predictive value for survival in patients with liver cirrhosis is significantly better and more reliable than that of traditional nutritional evaluation indicators like TSF thickness and BMI (18). In this study, the dry body weight BMI, TSF, grip strength and L3-SMI values of the observation group were higher than the baseline levels following the nutritional intervention. After 3 months of nutritional guidance, the L3-SMI value was higher in the observation group than in the control group. There was no statistical significance, which may have been related to factors such as the small sample size and the short observation time. The differences between the two groups before and after the intervention were further analyzed, and the results indicated that the increase in dry body weight BMI was higher in the observation group than in the control group. Overall, it was clear that the dietary guidance and intervention improved the skeletal muscle quality and function in varying degrees for the two groups of patients with hepatitis B cirrhosis.

Patients with liver cirrhosis often suffer from hypoalbuminemia due to insufficient intake, excessive body consumption, loss of body fluids and other factors that reduce the plasma colloid osmotic pressure and the circulating blood volume. This process can subsequently induce hepatic encephalopathy and hepatorenal syndrome, reducing the body's immune system resistance and increasing the possibility of infection. As the cirrhosis progresses, patients may experience progressive deterioration of liver function and the attendant signs and symptoms. In fact, most patients with cirrhosis experience multiple symptoms or complications, which can lead to a significant decrease in their quality of life (19). Previous studies have reported certain associations between quality of life and gastrointestinal symptoms, ascites, fatigue and other laboratory data in patients with cirrhosis; however, they have largely failed to consider the complications experienced by these patients and the degree of impairment of the hepatic reserve (20, 21).

The ALB, CHE, and pT values are clinical indicators that are commonly used to reflect the synthetic reserve function of the liver. In the current study, the patients in the observation group adhered to a nighttime snacking regime for 3 months. Following the intervention, the CHE value in this group was higher than the baseline level, while the ALB exhibited an improvement trend, albeit with no statistical significance. The control group adhered to a regular diet for 3 months. Following the intervention, the ALB in this group was slightly lower than the baseline level, and there was no significant difference. Moreover, the analysis of the ALB changes before and after the dietary guidance revealed that the level of ALB improvement was more pronounced in the observation group than in the control group.

Nighttime snacking can promote the recovery of the serum ALB and CHE levels in patients with hepatitis B cirrhosis. Long-term adherence to nighttime snacking can improve the liver function of such patients, reduce the supplementation of exogenous nutritional preparations and reduce medical costs.

In general, our current understanding of the energy metabolism process and nutritional intervention in patients with hepatitis B cirrhosis is limited. One of the issues that hindered the current study was the poor coordination of the patients. In addition, any cirrhosis patients who presented clear symptoms (itching, edema, ascites, etc.) were excluded from the study. A recent study of patients with cirrhosis suggested that emotional problems due to poor body self-image appear to be associated with poorer disease outcomes (22). When referring such patients for psychotherapy treatment, healthcare professionals should work to better understand the patients' physical self-images and guide them accordingly.

The limitations of this study include the short duration of the intervention and the small number of follow-up visits. Furthermore, since creatinine levels were not included in our initial data collection, Model For End Stage Liver Disease (MELD) scores were not available. This study did not detect this aspect of data, will be further studied in the future experiments. Meanwhile, the sample size was not sufficiently large for conducting further stratified research on patients with different liver function grades and severe complications. Further long-term and large-sample trials are thus needed to determine the ideal composition and effect of nighttime snacking.

## 5. Conclusion

The strengths of this study exist in the dietary guidance based on the participants' personal eating habits and food preferences, the understanding of the dietary pattern of nighttime snacking, the wide choice of food banks and the awareness that nighttime snacking is not restricted by location or living conditions. The results can be translated into clinical prescriptions for nutritional therapy in patients with hepatitis B cirrhosis. In addition, no serious adverse events related to the dietary intervention occurred in either group. Nighttime snacking interventions for hepatitis B cirrhosis patients with nutritional risks are beneficial in terms of the recovery of liver synthesis functions, the improvement in clinical indicators, sarcopenia corrections and the improvement in malnutrition-related complications.

## Data availability statement

The original contributions presented in the study are included in the article/Supplementary material, further inquiries can be directed to the corresponding author.

## Ethics statement

The studies involving human participants were reviewed and approved by the Second Hospital of Dalian Medical University. The patients/participants provided their written informed consent to participate in this study.

## Author contributions

Conception and design: ZH and RL. Provision of study materials or patients: ZZ. Data analysis and interpretation: YP. Administrative support and collection and assembly of data: RG. All authors contributed to the manuscript writing and final approval of manuscript.
