# Supplementary material for: Clinical effect of nighttime snacking on patients with hepatitis B cirrhosis
Source: Front Nutr. 2023 Jan 10;9:999462. doi: 10.3389/fnut.2022.999462 (PMC9871573; doi:10.3389/fnut.2022.999462)
Supplement: Supplementary file 1 [file Table_1.doc]

Supplement table 1. Remission of complications in observation group after 3 months of nighttime snack dietary guidance

| Types of complications | Before dietary guidance (*n*) | After dietary guidance (*n*) | Number of improved | |  |
| --- | --- | --- | --- | --- | --- |
| Portal vein emboli | 1 | 1 | | 0 | |
| Spontaneous bacterial peritonitis | 2 | 0 | | 2 | |
| Disturbance of electrolyte | 3 | 0 | | 3 | |
| gallbladder stones | 3 | 3 | | 0 | |
| Hepatic encephalopathy | 1 | 0 | | 1 | |
| Upper gastrointestinal bleeding | 1 | 0 | | 1 | |
| Small liver cancer | 4 | 4 | | 0 | |
