# Supplementary material for: Clinical effect of nighttime snacking on patients with hepatitis B cirrhosis
Source: Front Nutr. 2023 Jan 10;9:999462. doi: 10.3389/fnut.2022.999462 (PMC9871573; doi:10.3389/fnut.2022.999462)
Supplement: Supplementary file 2 [file Table_2.doc]

Supplement table 2. Remission of complications in control group after 3 months of nighttime snack dietary guidance

| Types of complications | Before dietary guidance (*n*) | After dietary guidance (*n*) | Number of improved |
| --- | --- | --- | --- |
| Liver and kidney syndrome | 1 | 1 | 0 |
| Spontaneous bacterial peritonitis | 2 | 1 | 1 |
| Disturbance of electrolyte | 3 | 3 | 0 |
| gallbladder stones | 3 | 3 | 0 |
| Hepatic encephalopathy | 3 | 2 | 1 |
| Upper gastrointestinal bleeding | 1 | 1 | 0 |
| Small liver cancer | 6 | 6 | 0 |
