# Supplementary material for: Clinical effect of nighttime snacking on patients with hepatitis B cirrhosis
Source: Front Nutr. 2023 Jan 10;9:999462. doi: 10.3389/fnut.2022.999462 (PMC9871573; doi:10.3389/fnut.2022.999462)
Supplement: Supplementary file 3 [file Table_3.doc]

Supplement table 3. GLIM diagnostic criteria related data.

| GLIM diagnostic procedure the first step: Patient's NRS-2002 score | | | | | GLIM diagnostic procedure the second step: the patient's phenotype and etiological type indicators | | | | |
| --- | --- | --- | --- | --- | --- | --- | --- | --- | --- |
|  |  |  |  |  | Indicators of phenotype | | | Etiological type index | |
| Group | Number | Age | Gender | NRS-2002 score | Weight loss | Lower BMI（＜18.5kg/m^2^） | Muscle mass（L3-SMI） | Reduced food intake or absorption | Disease burden/inflammatory state |
| Control group | 1 | 59 | male | 3 | Yes | 20.2 | 39.07 | Yes | Yes |
|  | 2 | 66 | male | 4 | Yes | 19.41 | 67.67 | Yes | Yes |
|  | 3 | 61 | male | 3 | No | 24.39 | 44.26 | Yes | Yes |
|  | 4 | 74 | female | 5 | Yes | 21.1 | 37.47 | Yes | Yes |
|  | 5 | 46 | male | 3 | No | 29.39 | 50.39 | Yes | Yes |
|  | 6 | 50 | male | 4 | No | 21.70 | 39.05 | Yes | Yes |
|  | 7 | 54 | male | 3 | Yes | 21.26 | 43.92 | Yes | Yes |
|  | 8 | 51 | male | 3 | Yes | 24.51 | 49.56 | Yes | Yes |
|  | 9 | 61 | male | 3 | Yes | 20.81 | 38.31 | Yes | Yes |
|  | 10 | 73 | female | 3 | Yes | 24.64 | 42.89 | Yes | Yes |
|  | 11 | 54 | male | 4 | Yes | 15.57 | 39.75 | Yes | Yes |
|  | 12 | 53 | male | 4 | Yes | 22.02 | 50.77 | Yes | Yes |
|  | 13 | 74 | male | 5 | No | 21.8 | 43.92 | Yes | Yes |
|  | 14 | 61 | male | 4 | Yes | 19.72 | 34.96 | Yes | Yes |
|  | 15 | 62 | female | 3 | No | 23.44 | 26.67 | Yes | Yes |
|  | 16 | 71 | male | 5 | Yes | 19.72 | 48.35 | No | No |
|  | 17 | 62 | female | 3 | Yes | 36.72 | 48.58 | Yes | Yes |
|  | 18 | 63 | female | 4 | No | 33.26 | 43.17 | Yes | Yes |
|  | 19 | 43 | male | 4 | No | 26.10 | 49.09 | Yes | Yes |
|  | 20 | 43 | male | 4 | Yes | 23.76 | 56.05 | Yes | Yes |
|  | 21 | 63 | female | 3 | Yes | 25.91 | 42.82 | Yes | Yes |
|  | 22 | 57 | male | 4 | No | 17.04 | 34.83 | Yes | Yes |
|  | 23 | 60 | male | 3 | Yes | 26.99 | 46.84 | No | Yes |
|  | 24 | 49 | male | 3 | Yes | 21.45 | 47.32 | Yes | Yes |
|  | 25 | 49 | female | 4 | Yes | 21.63 | 38.32 | Yes | Yes |
|  | 26 | 63 | female | 3 | No | 20.70 | 32.48 | Yes | Yes |
|  | 27 | 47 | male | 3 | Yes | 19.59 | 38.72 | Yes | Yes |
|  | 28 | 43 | male | 3 | Yes | 28.54 | 53.49 | Yes | Yes |
|  | 29 | 43 | male | 3 | Yes | 24.22 | 53.03 | Yes | Yes |
|  | 30 | 57 | female | 3 | Yes | 25.31 | 35.79 | No | Yes |
| Observation group | 1 | 71 | male | 5 | No | 32.10 | 48.56 | No | No |
|  | 2 | 66 | female | 4 | Yes | 21.7 | 34.73 | Yes | Yes |
|  | 3 | 54 | male | 3 | No | 22.0 | 46.34 | Yes | Yes |
|  | 4 | 34 | male | 3 | Yes | 21.6 | 48.69 | Yes | Yes |
|  | 5 | 47 | female | 4 | Yes | 24.61 | 40.30 | Yes | No |
|  | 6 | 63 | female | 3 | No | 33.78 | 50.68 | No | Yes |
|  | 7 | 65 | male | 3 | Yes | 20.9 | 45.64 | Yes | Yes |
|  | 8 | 69 | male | 3 | No | 22.6 | 32.56 | Yes | Yes |
|  | 9 | 44 | male | 3 | Yes | 25.1 | 55.75 | Yes | Yes |
|  | 10 | 73 | female | 4 | Yes | 24.6 | 42.90 | No | Yes |
|  | 11 | 56 | male | 4 | Yes | 24.7 | 53.29 | Yes | Yes |
|  | 12 | 44 | male | 4 | No | 30.1 | 56.41 | Yes | Yes |
|  | 13 | 62 | male | 3 | Yes | 27.0 | 52.04 | Yes | Yes |
|  | 14 | 67 | female | 5 | Yes | 21.6 | 29.49 | Yes | Yes |
|  | 15 | 26 | male | 3 | Yes | 18.8 | 43.38 | Yes | Yes |
|  | 16 | 46 | male | 3 | Yes | 29.4 | 57.96 | Yes | Yes |
|  | 17 | 61 | male | 3 | No | 29.1 | 53.51 | Yes | Yes |
|  | 18 | 62 | female | 4 | Yes | 22.6 | 40.34 | Yes | Yes |
|  | 19 | 47 | male | 3 | Yes | 20.9 | 50.26 | No | Yes |
|  | 20 | 34 | male | 3 | Yes | 28.3 | 55.77 | Yes | Yes |
|  | 21 | 55 | male | 3 | Yes | 18.7 | 42.74 | Yes | Yes |
|  | 22 | 59 | male | 4 | Yes | 18.7 | 44.74 | Yes | Yes |
|  | 23 | 68 | male | 3 | Yes | 26.1 | 45.00 | Yes | No |
|  | 24 | 49 | male | 3 | Yes | 22.0 | 48.46 | Yes | Yes |
|  | 25 | 70 | female | 4 | Yes | 24.1 | 33.18 | Yes | Yes |
|  | 26 | 45 | male | 3 | No | 24.1 | 45.82 | Yes | Yes |
|  | 27 | 67 | female | 3 | Yes | 23.0 | 40.09 | Yes | Yes |
|  | 28 | 38 | male | 3 | No | 24.7 | 45.45 | Yes | Yes |
|  | 29 | 46 | male | 3 | No | 25.2 | 46.38 | Yes | Yes |
|  | 30 | 57 | male | 4 | Yes | 20.16 | 33.1 | Yes | Yes |

Annotation: body mass index (BMI), Global Leadership Initiative on Malnutrition (GLIM), nutritional risk creening-2002 (NRS-2002).
