# Supplementary material for: Clinical effect of nighttime snacking on patients with hepatitis B cirrhosis
Source: Front Nutr. 2023 Jan 10;9:999462. doi: 10.3389/fnut.2022.999462 (PMC9871573; doi:10.3389/fnut.2022.999462)
Supplement: Supplementary file 4 [file Table_4.doc]

Supplement table 4. RFH-NPT related data.

| Group | Number | Age | Gender | Tube feeding nutrition | ascites | Whether fluid retention prevents the patient eating | BMI | Weight loss | Whether the patient's eating has been reduced by more than 1/2 in the past 5 days | Aggravated illness and/or no nutritional intake for more than 5 days | RFH-NPT score |
| --- | --- | --- | --- | --- | --- | --- | --- | --- | --- | --- | --- |
| Control group | 1 | 59 | male | No | mild | Yes | 20.2 | Yes | No |  | 4 |
|  | 2 | 66 | male | No | severe | Yes | 19.41 | Yes | Yes |  | 7 |
|  | 3 | 61 | male | No | No |  | 24.39 | No |  | No | 0 |
|  | 4 | 74 | female | No | moderate | Yes | 21.1 | Yes | No |  | 4 |
|  | 5 | 46 | male | No | No |  | 29.39 | No |  | Yes | 2 |
|  | 6 | 50 | male | No | mild | Yes | 21.70 | No | No |  | 3 |
|  | 7 | 54 | male | No | No |  | 21.26 | Yes |  | No | 1 |
|  | 8 | 51 | male | No | No |  | 24.51 | Yes |  | Yes | 4 |
|  | 9 | 61 | male | No | moderate | Yes | 20.81 | Yes | Yes |  | 7 |
|  | 10 | 73 | female | No | No |  | 24.64 | Yes |  | Yes | 3 |
|  | 11 | 54 | male | No | moderate | Yes | 15.57 | Yes | Yes |  | 7 |
|  | 12 | 53 | male | No | mild | Yes | 22.02 | Yes | No |  | 5 |
|  | 13 | 74 | male | Yes | moderate | Yes | 21.8 | No |  |  | 6 |
|  | 14 | 61 | male | No | mild | Yes | 19.72 | Yes | Yes |  | 7 |
|  | 15 | 62 | female | No | No |  | 23.44 | No |  | No | 0 |
|  | 16 | 71 | male | No | mild | No | 19.72 | Yes | No |  | 2 |
|  | 17 | 62 | female | No | No |  | 36.72 | Yes |  | Yes | 3 |
|  | 18 | 63 | female | No | moderate | Yes | 33.26 | No | No |  | 3 |
|  | 19 | 43 | male | No | mild | Yes | 26.10 | No | Yes |  | 5 |
|  | 20 | 43 | male | No | mild | Yes | 23.76 | Yes | No |  | 5 |
|  | 21 | 63 | female | No | No |  | 25.91 | Yes |  | No | 1 |
|  | 22 | 57 | male | No | severe | Yes | 17.04 | No | Yes |  | 5 |
|  | 23 | 60 | male | No | No |  | 26.99 | Yes |  | Yes | 3 |
|  | 24 | 49 | male | No | No |  | 21.45 | Yes |  | No | 2 |
|  | 25 | 49 | female | No | No |  | 21.63 | Yes |  | No | 2 |
|  | 26 | 63 | female | No | No |  | 20.70 | No |  | No | 0 |
|  | 27 | 47 | male | No | No |  | 19.59 | Yes |  | Yes | 5 |
|  | 28 | 43 | male | No | mild | Yes | 28.54 | Yes | No |  | 5 |
|  | 29 | 43 | male | No | No |  | 24.22 | Yes |  | No | 2 |
|  | 30 | 57 | female | No | mild | No | 25.31 | Yes | No |  | 1 |
| Observation group | 1 | 71 | male | Yes | mild | No | 32.10 | No |  |  | 6 |
|  | 2 | 66 | female | No | No |  | 21.7 | Yes |  | Yes | 3 |
|  | 3 | 54 | male | No | No |  | 22.0 | No |  | No | 0 |
|  | 4 | 34 | male | No | No |  | 21.6 | Yes |  | Yes | 3 |
|  | 5 | 47 | female | No | moderate | Yes | 24.61 | Yes | No |  | 5 |
|  | 6 | 63 | female | No | mild | Occasionally influence | 33.78 | No | No |  | 2 |
|  | 7 | 65 | male | No | mild | Yes | 20.9 | Yes | No |  | 5 |
|  | 8 | 69 | male | No | No |  | 22.6 | No |  | Yes | 2 |
|  | 9 | 44 | male | No | No |  | 25.1 | Yes |  | No | 1 |
|  | 10 | 73 | female | No | No |  | 24.6 | Yes |  | No | 2 |
|  | 11 | 56 | male | No | mild | Yes | 24.7 | Yes | No |  | 5 |
|  | 12 | 44 | male | No | No |  | 30.1 | No |  | No | 0 |
|  | 13 | 62 | male | No | No |  | 27.0 | Yes |  | Yes | 4 |
|  | 14 | 67 | female | No | severe | Yes | 21.6 | Yes | Yes |  | 7 |
|  | 15 | 26 | male | No | mild | Occasionally influence | 18.8 | Yes | Yes |  | 6 |
|  | 16 | 46 | male | No | No |  | 29.4 | Yes |  | No | 1 |
|  | 17 | 61 | male | No | No |  | 29.1 | No |  | No | 0 |
|  | 18 | 62 | female | No | severe | Yes | 22.6 | Yes | No |  | 5 |
|  | 19 | 47 | male | No | No |  | 20.9 | Yes |  | Yes | 4 |
|  | 20 | 34 | male | No | No |  | 28.3 | Yes |  | No | 1 |
|  | 21 | 55 | male | No | moderate | Yes | 18.7 | Yes | No |  | 5 |
|  | 22 | 59 | male | No | moderate | Yes | 18.7 | Yes | No |  | 5 |
|  | 23 | 68 | male | No | No |  | 26.1 | Yes |  | No | 1 |
|  | 24 | 49 | male | No | No |  | 22.0 | Yes |  | No | 2 |
|  | 25 | 70 | female | No | mild | Yes | 24.1 | Yes | No |  | 5 |
|  | 26 | 45 | male | No | No |  | 24.1 | No |  | No | 0 |
|  | 27 | 67 | female | No | No |  | 23.0 | Yes |  | No | 2 |
|  | 28 | 38 | male | No | No |  | 24.7 | No |  | No | 0 |
|  | 29 | 46 | male | No | No |  | 25.2 | No |  | Yes | 2 |
|  | 30 | 57 | male | No | severe | Yes | 20.16 | Yes | Yes |  | 7 |

Annotation: body mass index (BMI), Royal Free Hospital-Nutrition Prioritizing Tool (RFH-NPT).
